# Cross-Cultural Adaptation and Validation of the Turkish Form of the Awareness Scale on Consumption of Irradiated Foods (ASCIF)

**DOI:** 10.3390/foods15132278

**Published:** 2026-06-25

**Authors:** Demet Önen, Tiago Rusin, Wilma Maria Coelho Araújo

**Affiliations:** 1 Department of Nutrition and Dietetics, Faculty of Health Sciences, Gazi University, Ankara 06490, Turkey; demetonen@gazi.edu.tr; 2 Ministry of the Environment and Climate Change, Esplanada dos Ministérios, Block B, Brasília 70150-900, DF, Brazil; 3 College of Health Sciences, University of Brasília, Campus Darcy Ribeiro, Brasília 70910-900, DF, Brazil

**Keywords:** Turkish, food irradiation, perception, consumer, awareness, psychometric validation, cross-cultural adaptation, radura

## Abstract

Food irradiation is a well-established technology endorsed by the World Health Organization (WHO) to enhance food safety. However, consumer awareness of irradiated foods remains remarkably low across diverse cultural contexts. This study aimed to cross-culturally adapt and validate the Turkish form of the Awareness Scale for Consumption of Irradiated Foods (ASCIF). The ASCIF, originally developed and validated in Brazil, encompasses 31 items distributed across four factors: safety of irradiated foods (S), concepts (C), labeling (L), and awareness (A). The cross-cultural adaptation process adhered to the International Test Commission (ITC) guidelines, involving forward–backward translation and expert panel review. A total of 346 university-affiliated individuals (82.9% female; mean age 21.3 ± 4.8 years) from Gazi University, Ankara, completed an online survey with the adapted Turkish version of the ASCIF. Although the internal consistency results indicated a high level of reliability (α = 0.963), other indicators, such as ESEM analysis (RMSEA (90% CI) = 0.143 (0.139–0.148), CFI = 0.880, and TLI = 0.870), suggest that the cross-cultural adaptation of the ASCIF scale into Turkish encountered significant challenges, particularly regarding its psychometric validation. These complications indicate that the original construct may not have translated seamlessly across the cultural and linguistic nuances of the Turkish context in this study.

## 1. Introduction

Traditional thermal processing remains the dominant strategy in the food sector. These techniques encompass a range of applications, including pasteurization, sterilization, freezing, and adjusting pH or osmotic pressure. The primary goal of these methods is to ensure consumer safety by inhibiting the proliferation of spoilage and pathogenic microorganisms. However, such heat-based treatments can inadvertently degrade sensitive components, such as polyphenols and vitamins, thereby impacting the overall nutritional profile and quality of food [1,2].

As an alternative, food irradiation represents a well-established cold preservation method that improves sanitary quality through the elimination of parasites, reduction in bacterial loads, and control of insect infestations. Additionally, it serves physiological purposes in fresh commodities, such as delaying senescence and inhibiting sprouting [3]. The technique relies on exposing food matrices to controlled doses of ionizing radiation—namely gamma rays, X-rays, or accelerated electron beams—effectively destroying pathogens without rendering the food radioactive [4]. This process has maintained the official endorsement of the World Health Organization (WHO) since 1980 [5]. In Turkey, these practices are strictly governed by the Ministry of Agriculture and Forestry under the Food Irradiation Regulation. Irradiation is only permitted when there is a clear technological necessity and must never replace Good Manufacturing Practices (GMPs). Permissible sources include Cobalt-60 or Cesium-137 isotopes and electron beams or X-rays within specific energy thresholds. Turkish standards generally cap the absorbed dose at 10 kGy, in alignment with the Codex Alimentarius guidelines. Mandatory labeling is required; products must display the “Radura” symbol and phrases such as “Irradiated” to ensure transparency and consumer awareness. Turkey’s commercial irradiation infrastructure includes facilities in Ankara and Çerkezköy. While global leaders process millions of tons, Turkey’s output remains relatively modest, focusing primarily on spices, herbal teas, and dried vegetables for export [6].

While food irradiation scales globally, the Turkish socio-cultural and regulatory landscape presents distinct characteristics that differentiate it from the Latin American contexts where the ASCIF was originally validated. Structurally, although Turkey operates functional commercial gamma irradiation facilities in Ankara and Çerkezköy under the strict guidance of the Ministry of Agriculture and Forestry, the enforcement of mandatory ‘Radura’ and irradiation labeling remains communicatively invisible at the end-consumer level. This regulatory environment creates a paradox where a technology is legally authorized but remains practically unnoticeable to the public. Furthermore, Turkish consumers harbor a unique cultural script of ‘radiation-phobia’ deeply rooted in historical imagery, such as the post-Chernobyl legacy, which tends to conflate food processing with nuclear energy hazards. This specific public anxiety was partially documented by Gunes and Tekin (2006) but has not been psychometrically updated in the two decades since [7]. Consequently, evaluating whether a psychometric construct from Brazil or Argentina can transfer seamlessly to Turkey requires a dedicated cross-cultural exploration, filling a critical measurement gap for food safety stakeholders, dietetics educators, and regulatory institutions in the country [7]. Research on Turkish consumer behavior indicates significant hesitation, with approximately 80% of survey participants expressing uncertainty regarding safety [7]. However, studies suggest that providing clear information about the benefits can raise positive attitudes to 62%, particularly when prices remain competitive [7]. Similar patterns of skepticism and “radiation-phobia” have been observed in the Italian and Saudi Arabian contexts, where consumers often conflate irradiation with radioactivity [8,9].

To systematically evaluate these perceptions, a psychometric instrument termed the Awareness Scale on Consumption of Irradiated Foods (ASCIF) was developed and validated in Brazil using a diverse university sample that mirrored lay consumer behaviors [10]. The original validation confirmed a robust four-dimensional framework consisting of 31 items divided across distinct factors: safety of irradiated foods, concepts, labeling, and awareness. Characterized by high internal consistency, the ASCIF was proposed as an adaptable cross-cultural tool, offering a novel method to diagnose gaps in consumer knowledge and to predict market barriers or drivers for irradiated products [10]. In Argentina—where food irradiation is actively utilized but consumers frequently buy these items unconsciously due to poor labeling visibility and low baseline awareness—the ASCIF underwent its first international cross-cultural adaptation. Following the standard International Test Commission protocols, the adaptation process showed that most items required minimal changes (21 items classified as little changed), yielding an idiomatically appropriate Spanish version [11]. The psychometric stability of the Argentine scale was verified by an excellent Cronbach’s alpha coefficient of 0.988, establishing its transcultural utility [12]. Executing quantitative comparisons across diverse populations is highly facilitated by the deployment of psychometric metrics that have undergone cross-cultural validation. By accounting for international variations, this methodological approach aids in isolating critical determinants for designing successful strategic interventions. However, the scope of cross-cultural adaptation extends far beyond a simple semantic translation of an evaluative tool. It requires a holistic framework of adaptation and validation tailored to the specific sociological environment where deployment is intended. This pathway demands the precise handling of specialized terminology to build a functionally sound “target version” in the “target language” based on the original scale, relying on the active collaboration of bilingual experts and the maintenance of conceptual equivalence. Adhering to structured operational protocols developed and recommended by experienced investigators remains indispensable for investigations of this type [13]. The cross-cultural customization of assessment mechanisms, which balances linguistic conversion with targeted cultural modifications, plays an essential role in generating standardized, globally comparable data. This structural uniformity simplifies cross-study synthesis and provides a solid foundation for international multicenter research networks [12]. Within this field, the International Test Commission (ITC) offers a widely recognized and well-established framework for adapting psychometric scales [11]. According to the benchmarks set by the ITC, a literal textual translation constitutes merely an introductory component of a much broader adjustive sequence [11]. Adaptation serves as the overarching macro-concept, governing the systemic transfer of a test from its baseline linguistic and cultural ecosystem into an entirely separate population [11]. When transferring assessment scales, investigators can choose from multiple translation designs, including one-way direct translation, dual-panel approaches, or forward–backward translation protocols. The sequential forward and backward translation method stands out as the gold standard most frequently endorsed by international methodological guidelines. This configuration requires the participation of at least two independent language specialists acting blindly: the first translator converts the items into the designated “target language,” whereas the second translates this new draft back into the original “source language.” Empirical findings by Lee et al. indicate that both the forward–backward mechanism and the Dual-Panel strategy are highly capable of generating semantically equivalent translations; nonetheless, they emphasize that language translation alone cannot resolve deep cultural discrepancies [14]. Furthermore, Papadakis et al. compared translations produced by professionals with varying backgrounds and concluded that translators should ideally be bicultural, possess specialized domain knowledge regarding the instrument’s subject matter, and preferably be drawn from the target population [15]. To ascertain that an evaluation tool achieves comprehensive functional equivalence, investigators must systematically verify five distinct dimensions of stability. Conceptual fit represents the initial layer, which maps whether the underlying theoretical constructs and their mutual relationships hold actual relevance within the lifestyle and beliefs of the target population. Element alignment follows, demanding a rigorous scrutiny of the specific question parameters chosen to represent those theoretical domains. Semantic invariance is then required to guarantee that the newly translated phrasing accurately mirrors the original context, intent, and linguistic tone without introducing distortion. Operational suitability ensures that the execution protocols, media, and data collection settings are appropriate and practical for the local cultural environment. Psychometric invariance concludes the sequence by empirically testing the final data output to confirm that the scale’s statistical behavior and internal properties remain stable when compared to the baseline version. By successfully demonstrating each of these integrated parameters, researchers can confidently establish the global functional equivalence of the cross-culturally adapted scale [13,16]. These scientific toolkits—which commonly take the form of structured questionnaires, analytical tests, rating scales, and self-report inventories [17,18]—require a long and highly intricate validation trajectory. This process demands that researchers possess extensive technical expertise regarding translation logistics, back-translation verification, cultural adaptation, and pilot-testing parameters [13]. Investigators must also understand the underlying purposes and options of different psychometric properties, the empirical evidence required for their verification, and quantitative data processing using advanced statistical software [13]. Furthermore, these studies are vulnerable to specific risks of systematic bias that can distort the research process and compromise the validity of the final results [13].

A primary factor that researchers must fully comprehend prior to launching the translation, adaptation, and cross-cultural validation of any metric is the inherent risk of bias [11,13]. Socio-cultural biases represent a major threat to the integrity of this process [11,13]. Implementing proactive strategies—such as pre-testing the instrument with a pilot sample selected from the “target culture,” conducting cognitive debriefing interviews with respondents immediately following the pre-test, and utilizing standardized Likert-type response formats—serves as an effective mechanism to minimize these measurement errors [11,13].

Empirical psychometric validation represents a vital final phase within the overarching framework of cross-cultural adaptation. Regarding the selection of statistical protocols and design methodologies, extensive scholarly debate surrounds the exact sample sizes required to execute these validation stages. For instance, several researchers argue that sample adequacy should be determined by calculating statistical power thresholds, tracking missing data patterns, or prioritizing conceptual saturation over raw participant volume. Conversely, alternative benchmarks suggest that recruiting a cohort exceeding 100 respondents serves as an excellent operational standard for calculating measurement error, confirming reliability, executing hypothesis testing for construct validity, and evaluating subgroup differences. Similarly, some methodological guidelines recommend a target sample ranging from 100 to 200 individuals, while other psychometricians advocate for a minimum baseline of 200 participants. Furthermore, a widely utilized rule of thumb dictates maintaining a strict ratio of 10 respondents for every single item included in the evaluation tool. To assemble these study cohorts, investigators frequently employ non-probabilistic convenience sampling strategies, ensuring that the selected participants display demographic attributes that strictly align with the practical purpose of the metric [11,13,14,15]. Evaluating the structural integrity of an instrument involves analyzing diverse but interconnected validation benchmarks within psychometric literature. When examining scale reliability, methodological frameworks vary from established metrics of internal consistency—exemplified by Cronbach’s alpha—to temporal stability indicators derived from test–retest designs and quantified via Cohen’s coefficients or Intraclass Correlation Coefficients (ICCs). Methodological frameworks for structural validity and cross-cultural adaptation frequently rely on sequential factor analyses (EFA followed by CFA) or exploratory structural equation modeling (ESEM) to resolve complex latent structures. Furthermore, comprehensive validation requires rigorous construct and criterion evaluation, which involves verifying content relevance, dimensional stability, and measurement error, alongside empirical testing for convergent, discriminant, predictive, and concurrent validities through explicit hypothesis testing [11,12,13].

The ASCIF instrument was developed to assess awareness of the consumption of irradiated foods in Brasilia, Brazil. The instrument demonstrated good validity and internal reliability. This demonstrates its potential for adaptation to other languages and cultures. The data obtained reflect the profile of consumers of irradiated foods in the country, given that these foods are consumed unconsciously by most of the population [10].

During its empirical application within the Argentine context, data analysis satisfied all fundamental validation assumptions, confirming the structural robustness and reliability of the psychometric scale via Confirmatory Factor Analysis (CFA), Cronbach’s alpha coefficients, and Exploratory Structural Equation Modeling (ESEM). The final output successfully fulfilled all established benchmarks for internal consistency and construct validity evidence. Consequently, the instrument proved to be a highly efficient diagnostic tool for mapping market barriers and commercial drivers regarding the retail of irradiated products in Argentina, revealing that the majority of local consumers lacked baseline awareness regarding the practical benefits of this preservation technology [10,19]. Turkey was selected as the study population because it is a strategic agricultural hub with established gamma irradiation facilities in the country. However, there is a critical gap in validated psychometric tools for measuring Turkish consumer perceptions of this technology. This gap is highlighted by the clear cultural, developmental, and regulatory distinctions between Turkey and Latin American contexts (e.g., Brazil and Argentina), underscoring the need for a targeted cross-cultural adaptation of an instrument suitable for Turkish consumers.

To provide a rigorous theoretical anchor, the four ASCIF dimensions are situated within established consumer psychology frameworks. The Safety (S) factor maps onto the attitudinal constructs and outcome evaluations of the Theory of Planned Behavior [20], which mediate behavioral intentions to consume irradiated foods. The Concepts (C) factor aligns with Social Cognitive Theory [21], reflecting domain-specific knowledge acquisition through informational sources. Regulatory Trust Theory [22] underpins the Labeling (L) factor, capturing how institutional transparency and official symbols like the Radura predict novel technology acceptance. Lastly, the Awareness (A) factor mirrors the ‘unknown risk’ and ‘dread risk’ dimensions of the Psychometric Paradigm of Risk [23], illustrating that radiation-related emotional stigma operates independently of technical risk magnitudes. Consistent with values-related cross-cultural universality, these dimensions are expected to remain structurally stable while allowing for mean-level variations across distinct national populations.

Reflecting the high versatility of the ASCIF instrument for cross-cultural transposition, the primary purpose of this investigation was to execute the linguistic adaptation and psychometric validation of the Turkish version of the “Awareness Scale on Consumption of Irradiated Foods” (ASCIF) in a convenience sample consisting of individuals affiliated with Gazi University, including undergraduate and graduate students, faculty, and administrative staff.

## 2. Methodology

### 2.1. Cross-Cultural Adaptation of the ASCIF

Cross-cultural adaptation of the ASCIF involved the following steps: determining whether the test could validly assess the same underlying construct in the target language/culture as in the source; selecting qualified translators; adopting an evaluation design for translation quality (such as forward–backward translation); identifying required modifications or accommodations; altering test formats as needed; executing the translation; verifying equivalence in the new language and culture; and carrying out additional validity investigations.

To conduct the translation and backward translation stage of the ASCIF scale, five professionals proficient in both the Turkish and Portuguese languages, as well as Turkish culture, were invited. These experts performed a blind analysis independently without knowing one another, and all signed the Informed Consent Form (ICF). Any discrepancies that arose during the backward translation phase were thoroughly discussed and resolved by consensus, ensuring the conceptual equivalence and accuracy of the final instrument version.

A total of 18 specific recommendations partitioned across six operational dimensions compose the structural matrix of the ITC guidelines: pre-conditions (3), test development (5), confirmation through empirical analyses (4), administration (2), score scaling and interpretation (2), and documentation (2) [11]. Functioning as the foundational layer, the “Pre-Condition” phase highlights the critical necessity of settling fundamental administrative and structural choices prior to launching any textual translation or practical instrument adjustment [11]. The “Test Development Guidelines” category addresses the core procedures of test adaptation. The “Confirmation” category focuses on gathering empirical data to confirm equivalence, reliability, and validity across languages and cultures. The remaining three categories cover “Administration,” “Score Scales and Interpretation,” and “Documentation” [11]. To evaluate textual readability thresholds and accurately gauge the necessary completion time, the provisional Turkish draft was pilot-tested using a convenience sample of 20 individuals, specifically comprising university students and staff members.

### 2.2. Sample and Data Collection

The study sample (*n* = 346) comprised individuals affiliated with Gazi University, including undergraduate and graduate students, academic staff, and administrative personnel. Participants were recruited through convenience sampling using institutional email lists and online platforms. The eligibility criteria were as follows: (a) age ≥ 18 years, (b) current affiliation with the university, and (c) willingness to provide informed consent.

Data collection was executed electronically through a digital questionnaire interface, with the access link distributed across multiple networks to efficiently engage the target demographic. The assessment tool was structured into two main components: the first gathered sociodemographic and behavioral profiles, while the second presented the 31 items of the Turkish ASCIF scored on a 5-point Likert-type agreement matrix (1 = strongly disagree; 2 = disagree; 3 = neither agree nor disagree; 4 = agree; and 5 = strongly agree). Initially, a total of 483 respondents completed the online survey. To ensure data integrity, a rigorous screening protocol was applied to exclude problematic entries, such as individuals under 18 years of age, respondents who left entire sections of the psychometric scale blank, or those who provided contradictory answers that could not be transformed for processing. Following the removal of these incomplete or invalid cases from the initial 483 records, the final statistical analyses were conducted on a cleaned sample size of 346 participants.

### 2.3. Statistical Analysis

The selected statistical analyses were designed to validate the measurement tool specifically for the identified problem-solving strategies. To evaluate the structural and psychometric quality of the ASCIF Scale, Confirmatory Factor Analysis (CFA) was employed, while the overall reliability of the instrument was verified by calculating internal consistency metrics via Cronbach’s alpha. All data processing and statistical computations were executed using IBM SPSS (version 21) alongside JASP 0.97.0 software environments.

### 2.4. Ethical Approval

This cross-sectional investigation was carried out among cohorts affiliated with Gazi University in Ankara, Turkey. Official authorization for the study protocol was granted by the Gazi University Ethics Committee under Approval No. 2025-156 on 31 January 2025. Before any data collection commenced, electronic informed consent was secured from every individual participant. Furthermore, the operational and ethical execution of this research adhered strictly to the core principles established by the Declaration of Helsinki.

## 3. Results

### 3.1. Demographic Characteristics

In total, 483 individuals completed the survey (Supplementary File S1: ASCIF TR raw data). Following the exclusion of incomplete records, a final sample of 346 consumers participated in the study to execute the psychometric validation of the scale. The majority were female (*n* = 273, 78.9%), with a mean age of 21.4 ± 4.9 years. Undergraduate students constituted 92.2% of the sample (*n* = 319), and 95.4% were unmarried. With respect to eating behavior, 64.7% of the participants reported consuming two main meals daily, and 57.2% reported consuming one snack per day. Over half of the participants (52.9%) reported eating outside their homes daily. The complete demographic profiles are presented in Table 1.

### 3.2. Adaptation of Awareness Scale on Consumption of Irradiated Foods (ASCIF)

To execute this phase of the investigation, five specialists proficient in the Turkish and Portuguese languages, as well as thoroughly acquainted with Turkish culture, were selected in accordance with the methodological framework advocated by the International Test Commission (ITC) [11]. At the outset, each linguistic expert reviewed and signed an informed consent agreement. The adaptation process commenced with the forward translation of the instrument into Turkish, where an evaluation form featuring the original ASCIF items alongside dedicated translation fields was distributed. This initial step was carried out independently by two separate translators.

Once these two parallel forward translations were completed, two distinct subsequent forms compiling the Turkish adaptations were structured. These documents were then back-translated into the source language (Portuguese) by two different volunteer translators. Following this, a fifth independent translator, working in direct collaboration with the original author of the ASCIF, performed a comprehensive comparative synthesis of all generated versions against the cultural nuances of the target population to establish the definitive Turkish form of the ASCIF (Table 2).

Characteristically, the linguistic evaluators operated without any mutual contact, ensuring that both the forward- and back-translation stages were conducted in a strictly blinded and independent manner to safeguard the methodological rigor and objectivity of the cross-cultural adaptation. Table 2 details the systematically reviewed translations and back-translations, highlighting the semantic equivalence verified between the original Brazilian Portuguese instrument and the adapted Turkish version of the ASCIF.

### 3.3. Confirmatory Analysis of Data

#### 3.3.1. Analysis of Assumptions

Compliance with the foundational statistical assumptions of normality, linearity, and singularity was systematically evaluated. Data normality was verified through visual inspection of histogram distributions alongside the formal Shapiro–Wilk test. To validate the linearity hypothesis, residual scatterplots were examined, confirming that errors were randomly dispersed around the zero baseline. Finally, to assess the singularity criterion and rule out severe multicollinearity, the Variance Inflation Factor (VIF) and tolerance statistics were scrutinized; the diagnostic outputs demonstrated that VIF values fell within standard conservative boundaries and tolerance metrics consistently surpassed the 0.1 threshold, thereby confirming that the singularity assumption was satisfied [24,25,26].

#### 3.3.2. Confirmatory Factorial Analysis

The four-factor structure of the original ASCIF, comprising Safety of Irradiated Foods (S), Concepts (C), Labeling (L), and Awareness (A), was subjected to CFA using the item-rest correlation approach, which provides standardized factor loadings analogous to those obtained via maximum likelihood estimation. All factor loadings reached statistical significance (*p* < 0.05) and exceeded the conventional minimum threshold of 0.40, which is recommended for psychometric scale items. For Factor S (Safety; 15 items), the standardized loadings ranged from 0.638 (Q10: willingness to pay more for irradiated foods) to 0.815 (Q28: medium-term health safety), with a mean loading of 0.748 (SE range: 0.045–0.056). The mean item within this factor ranged from 2.801 (Q10) to 3.341 (Q9: would consume irradiated foods), indicating that willingness-to-pay and promotion-related items elicited the most neutral-to-cautious responses, whereas intention-to-consume items were rated slightly higher. For Factor C (Concepts; eight items), loadings spanned 0.532 (Q24: microbiological safety) to 0.750 (Q3: inhibition of sprouting), with means ranging from 3.361 (Q24) to 3.642 (Q7: shelf-life extension). For Factor L (Labeling; five items), loadings ranged from 0.687 (Q16: educational campaigns) to 0.843 (Q19: importance of additional label information), with item means between 3.633 and 3.766—the highest mean scores observed across the entire instrument, reflecting strong endorsement of transparency in food labeling. For Factor A (Awareness; three items), loadings were 0.692 (Q14), 0.635 (Q17), and 0.538 (Q8), with item means between 3.012 and 3.107. Notably, Q8 (“I consciously consume irradiated foods”) yielded both the lowest mean (3.095) and the lowest loading (0.538) across the scale, consistent with findings from prior validation studies in Brazil and Argentina, reflecting an absence of deliberate consumer choice regarding irradiated food. The full CFA output is presented in Table 3.

All inter-factor correlations were statistically significant (*p* < 0.001), providing evidence of convergent validity across the four dimensions of the ASCIF. The strongest association was observed between Safety (S) and Awareness (A) (r = 0.851), indicating a substantial conceptual overlap between safety perceptions and personal awareness of irradiated foods in the Turkish sample. The S–C (r = 0.665) and C–L (r = 0.696) correlations were moderate to high, suggesting that technical knowledge of irradiation (C) is meaningfully associated with both safety attitudes and labeling awareness. The S–L correlation (r = 0.499) was moderate, indicating that while safety perceptions and labeling sensitivity share common ground, they partially capture distinct constructs. The weakest correlation was found between Labeling (L) and Awareness (A) (r = 0.410), suggesting that these dimensions are the most discriminable pair within the instrument (Table 4).

#### 3.3.3. Reliability

To evaluate the internal consistency of the extracted dimensions, Cronbach’s alpha coefficients were calculated. The resulting reliability estimate reached a value of 0.963, demonstrating an exceptionally high degree of internal consistency and instrument stability. According to the benchmarks established by Taber [12], this value is classified as excellent, confirming the robustness of the measurement model. The total scale demonstrated excellent internal consistency (α = 0.963), consistent with the established benchmarks. At the subscale level, Factor S yielded α = 0.955, CR = 0.951, and AVE = 0.564; Factor L yielded α = 0.912, CR = 0.884, and AVE = 0.605; Factor C yielded α = 0.887, CR = 0.861, and AVE = 0.440; and Factor A yielded α = 0.778, CR = 0.655, and AVE = 0.391. Factors S and L met the α ≥ 0.70 and AVE ≥ 0.50 thresholds, thereby establishing strong evidence of convergent validity (Table 5).

Regarding the convergent validity of the scale, although the Average Variance Extracted (AVE) for the ‘Concepts’ and ‘Awareness’ dimensions fell below the ideal threshold of 0.50, their internal consistency and construct validity remained statistically supported. According to the widely accepted methodological criteria established by Fornell and Larcker [27], if the AVE is less than 0.50 but the Composite Reliability (CR) is higher than 0.60, the convergent validity of the construct is still considered adequate and psychometrically acceptable. For the present data, the CR values for these specific dimensions were well above the recommended thresholds (Concepts CR = 0.861; Awareness CR = 0.655). Therefore, despite the lower variance captured by the items alone, the strong internal reliability confirms the stability of these factors [27,28].

#### 3.3.4. Exploratory Structural Equation Modeling (ESEM)

**Figure 1:**
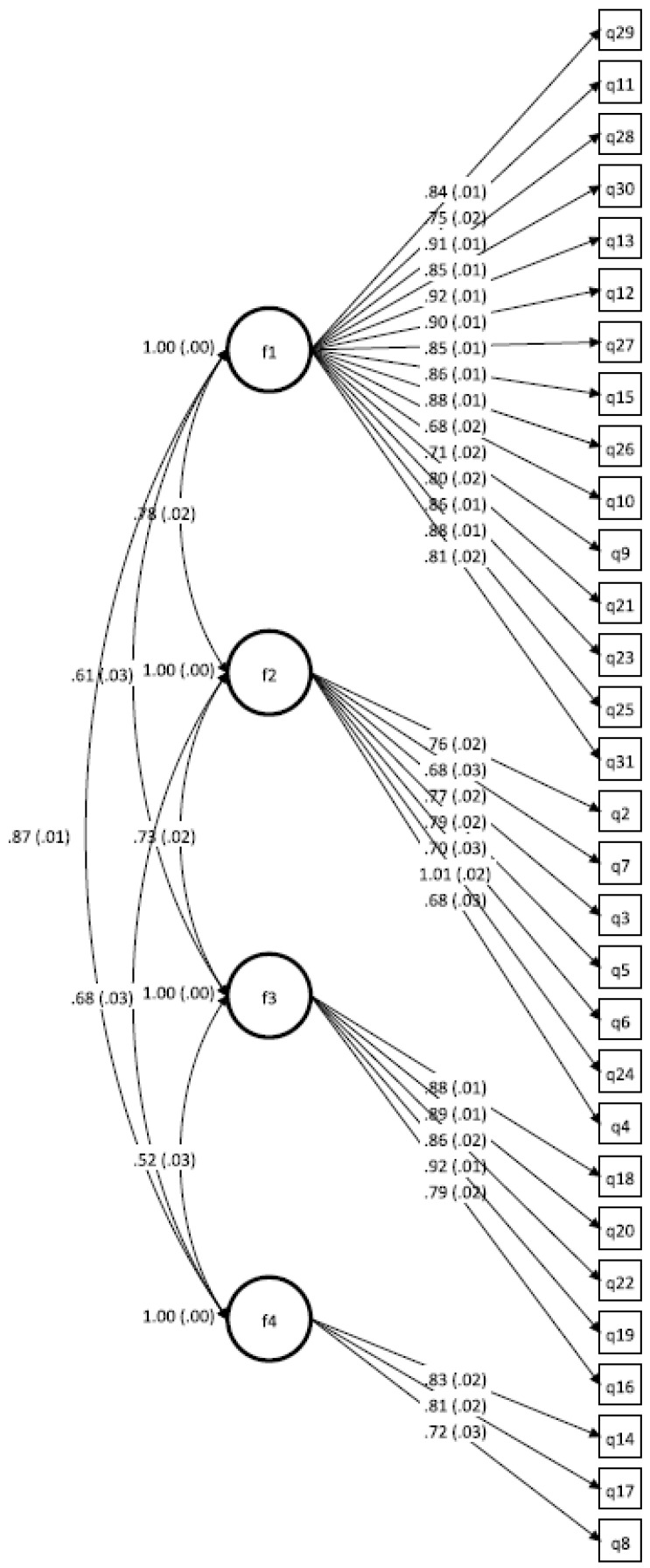
Structural model with StdYX coefficients.

To validate the structural framework against Confirmatory Factor Analysis (CFA) benchmarks, Structural Equation Modeling (SEM) was executed utilizing Mplus version 7 [29]. The global adequacy of the proposed factor architecture was monitored through a comprehensive suite of goodness-of-fit parameters, specifically the Root Mean Square Error of Approximation (RMSEA), the Comparative Fit Index (CFI), and the Tucker–Lewis Index (TLI). The empirical evaluation yielded an RMSEA (90% CI) of 0.143 (0.139–0.148), alongside a CFI of 0.880 and a TLI of 0.870. Within the Exploratory Structural Equation Modeling (ESEM) framework, the StdYX standardized coefficients demonstrated robust and stable loadings across the respective items and latent dimensions (Figure 1). However, the collective output of these global fit criteria implies that the initial ASCIF model exhibits a substandard fit regarding this specific dataset. Specifically, the RMSEA of 0.143, coupled with CFI and TLI parameters hovering below the 0.90 threshold, indicates that the model configuration does not align optimally with the data gathered from the 31 psychometric items measured on the 5-point Likert scale. These statistical indicators fell outside the conventional diagnostic cut-off boundaries established in psychometric literature, which generally mandate RMSEA values below 0.08 and CFI/TLI metrics exceeding 0.90 [30]. The CFI and TLI track the relative configuration of the target model against a restrictive baseline model; values exceeding 0.90 or ideally 0.95 traditionally signal satisfactory model-to-data alignment [31,32]. Conversely, the RMSEA quantifies the error of approximation per degree of freedom, where values below 0.05 denote a close fit and metrics up to 0.08 are deemed acceptable, even though this specific index heavily penalizes model complexity. In this investigation, the instrument’s RMSEA parameter (0.143) clearly diverged from acceptable structural thresholds. Importantly, this estimator is sensitive to and often penalizes highly complex multi-item frameworks [33], which characterizes the structural design of the model tested here. Lastly, under alternative comparative lenses, the CFI and TLI approached baseline parameters by exceeding 0.85, yet remained below optimal thresholds. Consequently, the initial ASCIF framework revealed structural vulnerabilities regarding its direct applicability within the Turkish cultural context.

### 3.4. Potential Applications

Pending broader representative validation, the ASCIF-TR holds exploratory utility for preliminary assessment purposes, provided that its current psychometric boundaries are carefully considered. In education, it can function as a diagnostic tool for identifying specific knowledge gaps among different population segments, thereby informing the design of curricula and continuing education programs in nutrition, food science and public health. From a policy perspective, baseline data from the Turkish ASCIF can support regulatory bodies, such as the Ministry of Agriculture and Forestry, in evaluating the effectiveness of food irradiation labeling regulations and designing evidence-based public awareness campaigns. For the industry, understanding consumer perceptions through a psychometrically validated instrument can guide marketing strategies and consumer communication approaches for irradiated food products.

Despite the localized psychometric challenges observed during cross-cultural adaptation, the Turkish version of the ASCIF scale offers valuable exploratory utility and provides a foundational framework for future research. The observed validation challenges, likely exacerbated by the limited subject-to-item ratio and the profound linguistic divergence between Portuguese and Turkish, underscore the complexity of measuring food irradiation awareness across distinct cultural landscapes. Nevertheless, the instrument serves as a critical exploratory tool for mapping domain literacy gaps and identifying cultural barriers to food technology acceptance. These findings provide essential preliminary evidence for tailoring public health communication strategies and refining food labeling regulations, while also offering a baseline for the future development of culturally nuanced and psychometrically optimized measurement models within the Turkish food industry.

### 3.5. Limitations

It is important to acknowledge that the educational levels in Turkey vary significantly across demographic groups. While the sample in this study reflects a highly educated profile, lower educational levels are generally more prevalent in rural regions. As a result, the representation of university-educated individuals and active students within this sample exceeded nationwide demographic proportions, a factor that potentially restricts the immediate generalizability of these insights to the wider Turkish populace. To address this limitation, subsequent investigations should prioritize enhancing sample diversity and expanding the overall sociodemographic spectrum of participants. The Turkish version of the ASCIF revealed structural divergences from the original Brazilian model. Construct validity through CFA indicated that some items within the ‘Concepts’ and ‘Safety’ factors showed cross-loadings and lower variance extraction, suggesting a conceptual overlap in the Turkish cultural perception of food technology, safety, and radioactivity.

The demographic profile of this study, characterized by a significant female proportion (78.9%) and a young academic population (mean age 21.4 years), provides a strategic perspective on the future of the Turkish food industry. Traditionally, women are the primary decision-makers regarding household food choices and safety. The observed skepticism and challenges in validating the ‘Safety’ factor within this group suggest that even high levels of education do not automatically mitigate radiation-phobia. This indicates that food irradiation acceptance in Turkey is deeply rooted in emotional and cultural perceptions, rather than purely technical knowledge.

Nevertheless, these outcomes must be interpreted with caution due to certain methodological constraints. Primarily, the sample size ratio—comprising approximately eight respondents per individual psychometric item—presents a potential limitation regarding the global statistical power and the subsequent generalizability of the empirical findings derived from this study. Furthermore, the cross-cultural adaptation of the ASCIF from Portuguese to Turkish introduced significant linguistic and contextual nuances that may have influenced respondents’ interpretation. Finally, the Turkish cohort’s lack of foundational knowledge of food irradiation could have skewed the data, as participants likely relied on intuitive risk perceptions rather than technical understanding.

## 4. Conclusions

In conclusion, the findings demonstrate that the Turkish form of the ASCIF (ASCIF-TR) possesses acceptable psychometric properties and can be employed as a valid instrument for assessing consumer awareness of irradiated foods. Although the data were collected from a study group with a high education level, this demographic represents an important segment of adult consumers whose perspectives offer valuable insights into food safety literacy. Therefore, the ASCIF-TR serves as a robust tool for evaluating food irradiation awareness in the Turkish language. To further build upon these findings, future research incorporating larger samples with a broader range of sociodemographic and educational backgrounds could be conducted to evaluate the scale’s wider generalizability. The findings confirm that, despite Turkey’s pioneering role in the commercial application of food irradiation, awareness among the university-affiliated population remains moderate, with consumers expressing particular uncertainty about their conscious consumption of irradiated products and reluctance to pay a premium for such products. Simultaneously, the strong endorsement of labeling-related items points to a clear consumer demand for greater transparency. These results contribute to the growing international evidence base supporting the cross-cultural applicability of the ASCIF and highlight the need for evidence-based educational interventions tailored to the Turkish population’s needs.

In conclusion, while the cross-cultural adaptation followed rigorous ITC guidelines, the psychometric validation indicated that ‘awareness’ is a culturally dependent construct. The Turkish population (specifically, academic youth) exhibits a high level of skepticism that merges safety concerns with technical misconceptions. For food authorities in Turkey, this study highlights that providing information alone is insufficient, and communication strategies must decouple food irradiation from nuclear energy myths. Future research should refine ASCIF items to better capture the nuances of Middle Eastern consumer behavior. Future studies should also extend the validation to nationally representative samples, incorporate confirmatory factor analysis, and evaluate the longitudinal sensitivity of the instrument to changes in consumer awareness following educational campaigns.

## Figures and Tables

**Table 1:** Sociodemographic profiles of surveyed participants (*n* = 346).

Demographic Variables	*n*	%
**Gender**		
Female	273	78.9
Male	73	21.1
**Age (years)**		
18–20	127	36.7
21–25	166	48.0
26–54	53	15.3
Mean ± SD		21.4 ± 4.9
**Education Level**		
Undergraduate student	319	92.2
Bachelor’s degree	18	5.2
Postgraduate degree	9	2.6
**Marital Status**		
Single	330	95.4
Married	16	4.6

**Table 2:** Conceptual and Semantic Correspondence Between the Source Portuguese Instrument and the Adapted Turkish Version of the ASCIF.

Original ASCIF [10] (Portuguese)	Version 1			Version 2			Final Version
Original Version [10]	Translation 1	Retranslation 1	Version 1 Assessment *	Translation 2	Retranslation 2	Version 2 Assessment *	Comparison Between Versions and Turkish Culture.
Q1. Alimento irradiado é diferente de alimento radioativo. (Q1. Irradiated food is different from radioactive food.)	Q1. Gıdaların ışınlanması ile radyoaktif olması farklı şeylerdir.	Q1. Irradiação de alimentos e radioatividade são duas coisas diferentes.	LC	Q1. Işınlanan gıdalar radyoaktif gıdalardan farklıdır.	Q1. Os alimentos irradiados são diferentes dos alimentos radioativos.	UC	Q1. Gıdaların ışınlanması ile radyoaktif olması farklı şeylerdir. (Q1. Irradiated food is different from radioactive food.)
Q2. A irradiação de alimentos pode ser utilizada para reduzir a carga microbiana em alimentos. (Q2. Food irradiation can be used to reduce the microbial load on food.)	Q2. Gıda ışınlama, gıdalardaki mikrobiyal yükü azaltmak için kullanılabilir.	Q2. A irradiação de alimentos pode ser usada para reduzir a carga microbiana nos alimentos.	UC	Q2. Gıdaların ışınlanması, gıdaların üstündeki mikrobiyal yükü azaltmak için kullanılabilir.	Q2. A irradiação de alimentos pode ser usada para reduzir a carga microbiana nos alimentos.	UC	Q2. Gıda ışınlama, gıdalardaki mikrobiyal yükü azaltmak için kullanılabilir. (Q2. Food irradiation can be used to reduce the microbial load on food.)
Q3. A irradiação de alimentos pode ser utilizada para inibir o brotamento de bulbos, raízes e tubérculos. (Q3. The irradiation of food can be used to inhibit the budding of bulbs, roots and tubers.)	Q3. Gıda ışınlama, soğan, kök ve yumruların filizlenmesini engellemek için kullanılabilir.	Q3. A irradiação de alimentos pode ser usada para evitar o brotamento de cebolas, raízes e tubérculos.	LC	Q3. Işınlanmış gıdalar soğanların (ampullerin), köklerin ve yumruların filizlenmesini engellemek için kullanılabilir.	Q3. Os alimentos irradiados podem ser usados para evitar que cebolas (bulbos), raízes e tubérculos brotem.	LC	Q3. Gıda ışınlama, soğan, kök ve yumruların filizlenmesini engellemek için kullanılabilir. (Q3. The irradiation of food can be used to inhibit the budding of bulbs, roots and tubers.)
Q4. A irradiação de alimentos pode ser utilizada para adiar/retardar o amadurecimento de frutas. (Q4. Food irradiation can be used to delay the ripening of fruits.)	Q4. Gıda ışınlama, meyvelerin olgunlaşmasını geciktirmek/yavaşlatmak için kullanılabilir.	Q4. A irradiação de alimentos pode ser usada para atrasar/retardar o amadurecimento de frutas.	UC	Q4. Gıdaların ışınlanması meyvelerin olgunlaşmasını ertelemek veya geciktirmek için kulanılabilir.	Q4. A irradiação de alimentos pode ser usada para retardar ou adiar o amadurecimento de frutas.	UC	Q4. Gıda ışınlama, meyvelerin olgunlaşmasını geciktirmek/yavaşlatmak için kullanılabilir. (Q4. Food irradiation can be used to delay the ripening of fruits.)
Q5. A dose mínima absorvida pelo alimento irradiado deve ser suficiente para alcançar a finalidade pretendida. (Q5. The minimum absorbed dose by the irradiated food must be sufficient to achieve the intended purpose.)	Q5. Işınlanan gıdanın emdiği minimum doz, amaçlanan etkiyi sağlamak için yeterli olmalıdır.	Q5. A dose mínima absorvida pelo alimento irradiado deve ser suficiente para atingir o efeito pretendido.	LC	Q5. Işınlanmış gıda tarafından absorbe (emilen) minimum doz, hedeflenen amacı başarmak için yeterli olmalıdır.	Q5. A dose mínima absorvida pelo alimento irradiado deve ser suficiente para atingir o objetivo pretendido.	UC	Q5. Işınlanan gıdanın emdiği minimum doz, amaçlanan etkiyi sağlamak için yeterli olmalıdır. (Q5. The minimum absorbed dose by the irradiated food must be sufficient to achieve the intended purpose.)
Q6. O Brasil autoriza o uso da irradiação de alimentos. (Q6. Brazil authorizes the use of food irradiation.)	Q6. Türkiye, gıdaların ışınlanmasına izin vermektedir.	Q6. O Brasil autoriza a irradiação de alimentos.	LC	Q6. Türkiye, gıda ışınlanmasının kullanımına izin veriyor.	Q6. O Brasil autoriza o uso da irradiação de alimentos.	UC	Q6. Türkiye, gıdaların ışınlanmasına izin vermektedir. (Q6. Türkiye authorizes the use of food irradiation.)
Q7. A irradiação de alimentos pode ser utilizada para aumentar a vida útil ou o prazo de validade dos alimentos. (Q7. Food irradiation can be used to increase shelf life.)	Q7. Gıda ışınlama, gıdaların raf ömrünü veya son kullanma tarihini uzatmak için kullanılabilir.	Q7. A irradiação de alimentos pode ser usada para estender a vida útil ou a data de validade dos alimentos.	UC	Q7. Gıda ışınlanması, gıdaların son kullanma tarihini veya kullanım ömrünü (raf ömrü) arttırmak için kullanılabilir.	Q7. A irradiação de alimentos pode ser usada para aumentar a data de validade ou a vida útil (prazo de validade) dos alimentos.	UC	Q7. Gıda ışınlama, gıdaların raf ömrünü veya son kullanma tarihini uzatmak için kullanılabilir. (Q7. Food irradiation can be used to increase shelf life.)
Q8. Eu consumo conscientemente alimentos irradiados. (Q8. I consciously consume irradiated food.)	Q8. Işınlanmış gıdaları bilinçli olarak tüketiyorum.	Q8. Eu consumo alimentos irradiados conscientemente.	UC	Q8. Işınlanmış gıdaları bilinçli olarak tüketiyorum.	Q8. Eu consumo alimentos irradiados conscientemente.	UC	Q8. Işınlanmış gıdaları bilinçli olarak tüketiyorum. (Q8. I consciously consume irradiated food.)
Q9. Eu consumiria alimentos irradiados. (Q9. I would consume irradiated food.)	Q9. Işınlanmış gıdaları tüketirim.	Q9. Eu consumo alimentos irradiados.	LC	Q9. Işınlanmış gıdaları tüketirdim.	Q9. Eu costumava comer alimentos irradiados.	VC	Q9. Işınlanmış gıdaları tüketirim. (Q9. I would consume irradiated food.)
Q10. Eu estaria disposto a pagar mais por alimentos irradiados. (Q10. I would be willing to pay more for irradiated food.)	Q10. Işınlanmış gıdalar için daha fazla ödeme yapmaya istekliyim.	Q10. Estou disposto a pagar mais por alimentos irradiados.	LC	Q10. Işınlanmış gıdalar için daha fazla ödemek için istekli olurdum.	Q10. Eu estaria disposto a pagar mais por alimentos irradiados.	UC	Q10. Işınlanmış gıdalar için daha fazla ödeme yapmaya istekliyim. (Q10. I would be willing to pay more for irradiated food.)
Q11. Eu incentivaria o consumo de alimentos irradiados. (Q11. I would encourage consumption of irradiated foods.)	Q11. Işınlanmış gıdaların tüketimini teşvik ederim.	Q11. Eu incentivo o consumo de alimentos irradiados.	UC	Q11. Işınlanmış gıdaların tüketimini teşvik ederdim.	Q11. Eu incentivaria o consumo de alimentos irradiados.	UC	Q11. Işınlanmış gıdaların tüketimini teşvik ederim. (Q11. I would encourage consumption of irradiated foods.)
Q12. Eu consumiria alimentos irradiados, pois sei que estes não causam danos à saúde. (Q12. I would consume irradiated foods, as I know they do not cause health damage.)	Q12. Işınlanmış gıdaları tüketirim çünkü bunların sağlığa zarar vermediğini biliyorum.	Q12. Eu consumo alimentos irradiados porque sei que eles não são prejudiciais à saúde.	UC	Q12. Işınlanmış gıdaların sağlığa zarar vermediğini bildiğim için tüketeceğim.	Q12. Eu consumirei alimentos irradiados porque sei que eles não prejudicam a saúde.	UC	Q12. Işınlanmış gıdaları tüketirim çünkü bunların sağlığa zarar vermediğini biliyorum. (Q12. I would consume irradiated foods, as I know they do not cause health damage.)
Q13. Eu consumiria alimentos irradiados, pois sei que estes são seguros para o consumo. (Q13. I would consume irradiated food because I know that these are safe for consumption.)	Q13. Işınlanmış gıdaları tüketirim çünkü bunların güvenli olduğunu biliyorum.	Q13. Eu consumo alimentos irradiados porque sei que são seguros.	LC	Q13. Işınlanmış gıdaların tüketiminin (sağlıklı) güvenli olduğunu bildiğimden tüketirdim.	Q13. Eu consumiria alimentos irradiados porque sei que seu consumo é (saudável) seguro.	LC	Q13. Işınlanmış gıdaları tüketirim çünkü bunların güvenli olduğunu biliyorum. (Q13. I would consume irradiated food because I know that these are safe for consumption.)
Q14. Eu conheço algum alimento irradiado. (Q14. I know some irradiated food.)	Q14. Işınlanmış bir gıda ürünü biliyorum.	Q14. Conheço um produto alimentício irradiado.	LC	Q14. Ben bazı ışınlanmış gıdaları bilirim.	Q14. Conheço alguns alimentos irradiados.	UC	Q14. Işınlanmış bir gıda ürünü biliyorum. (Q14. I know some irradiated food.)
Q15. Eu aprovo o consumo de alimentos irradiados. (Q15. I approve of the consumption of irradiated foods.)	Q15. Işınlanmış gıdaların tüketimini onaylıyorum.	Q15. Aprovo o consumo de alimentos irradiados.	UC	Q15. Işınlanmış gıdaların tüketimini onaylıyorum.	Q15. Aprovo o consumo de alimentos irradiados.	UC	Q15. Işınlanmış gıdaların tüketimini onaylıyorum. (Q15. I approve of the consumption of irradiated foods.)
Q16. Eu considero ser necessário fazer campanhas educativas para informar a população sobre a irradiação de alimentos. (Q16. I consider it necessary to carry out educational campaigns to inform the population about the irradiation of food.)	Q16. Halkı gıda ışınlama konusunda bilgilendirmek için eğitim kampanyalarının gerekli olduğunu düşünüyorum.	Q16. Acredito que campanhas educativas são necessárias para informar o público sobre a irradiação de alimentos.	LC	Q16. Işınlanmış gıda konusunda halkı bilgilendirmek için eğitim kampanyalarının yapılmasının ihtiyaç olduğunu düşünüyorum.	Q16. Acho que há necessidade de campanhas educacionais para informar o público sobre alimentos irradiados.	VC	Q16. Halkı gıda ışınlama konusunda bilgilendirmek için eğitim kampanyalarının gerekli olduğunu düşünüyorum. (Q16. I consider it necessary to carry out educational campaigns to inform the population about the irradiation of food.)
Q17. Eu conheço a Radura, símbolo utilizado para representar um alimento irradiado. (Q17. I know Radura, the symbol used to represent irradiated food.)	Q17. Işınlanmış bir gıdayı temsil eden “Radura” sembolünü biliyorum.	Q17. Conheço o símbolo “Radura”, que representa um alimento irradiado.	UC	Q17. Işınlanmış bir gıdayı temsil etmek için kullanılan bir sembol olan Radura’yı biliyorum.	Q17. Eu conheço o Radura, um símbolo usado para representar um alimento irradiado.	LC	Q17. Işınlanmış bir gıdayı temsil eden “Radura” sembolünü biliyorum. (Q17. I know Radura, the symbol used to represent irradiated food.)
Q18. Todos os alimentos que passam por processo de irradiação deveriam ter essa informação destacada no rótulo do produto. (Q18. All foods that undergo irradiation should have this information highlighted on the product label.)	Q18. Işınlama işleminden geçen tüm gıdaların ambalajında bu bilginin açıkça belirtilmesi gerektiğini düşünüyorum.	Q18. Acho que essa informação deve ser claramente indicada na embalagem de todos os alimentos irradiados.	VC	Q18. Işınlanma sürecinden geçen tüm gıdaların etiketlerinin üzerinde bu bilginin olması gereklidir.	Q18. Todos os alimentos que foram submetidos à irradiação devem ter essa informação em seus rótulos.	LC	Q18. Işınlama işleminden geçen tüm gıdaların ambalajında bu bilginin açıkça belirtilmesi gerektiğini düşünüyorum. (Q18. All foods that undergo irradiation should have this information highlighted on the product label.)
Q19. Eu considero que as informações adicionais contidas nos rótulos dos alimentos irradiados são importantes. (Q19. I consider that the additional information contained in the labels of irradiated foods is important.)	Q19. Işınlanmış gıdaların etiketlerindeki ek bilgilerin önemli olduğunu düşünüyorum.	Q19. Acho que informações adicionais sobre a rotulagem de alimentos irradiados são importantes.	UC	Q19. Işınlanmış gıdalara eklenen ek bilgilerin önemli olduğunu düşünüyorum.	Q19. Acho que as informações adicionais adicionadas aos alimentos irradiados são importantes.	LC	Q19. Işınlanmış gıdaların etiketlerindeki ek bilgilerin önemli olduğunu düşünüyorum. (Q19. I consider that the additional information contained in the labels of irradiated foods is important.)
Q20. Eu considero importante o símbolo da Radura nos rótulos dos alimentos irradiados. (Q20. I consider the symbol of Radura important in the labels of irradiated foods.)	Q20. Işınlanmış gıdaların etiketlerinde Radura sembolünün olmasının önemli olduğunu düşünüyorum.	Q20. Acho que é importante ter o símbolo da Radura nos rótulos dos alimentos irradiados.	UC	Q20. Işınlanmış gıdaların üzerindeki Radura etiketinin önemli olduğunu düşünüyorum.	Q20. Acho que o rótulo Radura nos alimentos irradiados é importante.	VC	Q20. Işınlanmış gıdaların etiketlerinde Radura sembolünün olmasının önemli olduğunu düşünüyorum. (Q20. I consider the symbol of Radura important in the labels of irradiated foods.)
Q21. Eu tenho segurança em comprar um alimento quando leio no rótulo a seguinte informação “alimento tratado por processo de irradiação”. (Q21. I have confidence in buying a food when I read on the label the following information “food treated by irradiation process”.)	Q21. Bir gıdanın etiketinde “ışınlama işlemiyle muamele edilmiştir” ifadesini okuduğumda o gıdayı güvenle satın alırım.	Q21. Quando leio a frase “tratado por irradiação” no rótulo de um alimento, compro esse alimento com confiança.	LC	Q21. Bir gıdanın etiketi üzerinde ışınlanma sürecinden geçmiş bilgisini okuduğumda, satın alırken kendime güveniyorum.	Q21. Quando leio no rótulo de um alimento que passou pelo processo de irradiação, sinto-me confiante ao comprá-lo.	VC	Q21. Bir gıdanın etiketinde “ışınlama işlemiyle muamele edilmiştir” ifadesini okuduğumda o gıdayı güvenle satın alırım. (Q21. I have confidence in buying a food when I read on the label the following information “Food Treated with Ionizing Energy”.)
Q22. O rótulo dos alimentos deveria destacar a informação de alimento irradiado. (Q22. The food label should highlight the information of irradiated food.)	Q22. Gıda etiketlerinde “ışınlanmış gıda” bilgisinin öne çıkarılması gerektiğini düşünüyorum.	Q22. Acho que as informações sobre “alimentos irradiados” devem ser enfatizadas nos rótulos dos alimentos.	LC	Q22. Gıda etiketleri ışınlanmış gıda bilgisini belirtmelidir/vurgulanmalıdır.	Q22. Os rótulos dos alimentos devem indicar/enfatizar as informações sobre alimentos irradiados.	LC	Q22. Gıda etiketlerinde “ışınlanmış gıda” bilgisinin öne çıkarılması gerektiğini düşünüyorum. (Q22. The food label should highlight the information of irradiated food.)
Q23. Eu compraria alimentos irradiados, pois sei que este processo não torna o alimento radioativo. (Q23. I would buy irradiated food because I know this process does not make the food radioactive.)	Q23. Işınlanmış gıdaları satın alırım çünkü bu işlemin gıdayı radyoaktif hale getirmediğini biliyorum.	Q23. Eu compro alimentos irradiados porque sei que esse processo não torna o alimento radioativo.	UC	Q23. Bu işlemin radyoaktif gıdaya dönüştürdüğünü bildiğim için ışınlanmış gıdayı alırdım.	Q23. Eu compraria alimentos irradiados porque sei que esse processo os transforma em alimentos radioativos.	CC	Q23. Işınlanmış gıdaları satın alırım çünkü bu işlemin gıdayı radyoaktif hale getirmediğini biliyorum. (Q23. I would buy irradiated food because I know this process does not make the food radioactive.)
Q24. Os alimentos irradiados são seguros sob o aspecto microbiológico. (Q24. Irradiated food is microbiologically safe.)	Q24. Işınlanmış gıdalar mikrobiyolojik açıdan güvenlidir.	Q24. Os alimentos irradiados são microbiologicamente seguros.	UC	Q24. Işınlanmış gıdalar mikrobiyolojik yönden güvenlidir.	Q24. Os alimentos irradiados são microbiologicamente seguros.	UC	Q24. Işınlanmış gıdalar mikrobiyolojik açıdan güvenlidir. (Q24. Irradiated food is microbiologically safe.)
Q25. Os alimentos irradiados são seguros sob o aspecto nutricional. (Q25. Irradiated foods are nutritionally safe.)	Q25. Işınlanmış gıdalar besin değeri açısından güvenlidir.	Q25. Os alimentos irradiados são seguros em termos de valor nutricional.	UC	Q25. Işınlanmış gıdalar besin yönünden güvenlidir.	Q25. Os alimentos irradiados são nutricionalmente seguros.	LC	Q25. Işınlanmış gıdalar besin değeri açısından güvenlidir. (Q25. Irradiated foods are nutritionally safe.)
Q26. Eu me sinto seguro quanto ao consumo de alimentos irradiados. (Q26. I feel safe about the consumption of irradiated foods.)	Q26. Işınlanmış gıdaları tüketme konusunda kendimi güvende hissediyorum.	Q26. Sinto-me seguro para consumir alimentos irradiados.	UC	Q26. Işınlanmış gıda tükettiğimde, kendimi güvende hissediyorum.	Q26. Quando consumo alimentos irradiados, sinto-me seguro.	LC	Q26. Işınlanmış gıdaları tüketme konusunda kendimi güvende hissediyorum. (Q26. I feel safe about the consumption of irradiated foods.)
Q27. Eu considero que os alimentos irradiados não fazem mal à saúde a curto prazo. (Q27. I consider that irradiated foods are not harmful to health in the short term.)	Q27. Işınlanmış gıdaların kısa vadede sağlığa zarar vermediğini düşünüyorum.	Q27. Acredito que os alimentos irradiados não prejudicam a saúde em curto prazo.	UC	Q27. Işınlanmış gıdaların kısa vadede sağlığa zarar vermeyeceğini düşünüyorum.	Q27. Acredito que os alimentos irradiados não prejudicarão a saúde em curto prazo.	LC	Q27. Işınlanmış gıdaların kısa vadede sağlığa zarar vermediğini düşünüyorum. (Q27. I consider that irradiated foods are not harmful to health in the short term.)
Q28. Eu considero que os alimentos irradiados não fazem mal à saúde a médio prazo. (Q28. I consider that irradiated foods are not harmful to health in the medium term.)	Q28. Işınlanmış gıdaların orta vadede sağlığa zarar vermediğini düşünüyorum.	Q28. Acredito que os alimentos irradiados não prejudicam a saúde em médio prazo.	UC	Q28. Orta vadede ışınlanmış gıdaların sağlığa zarar vermeyeceğini düşünüyorum.	Q28. Acredito que os alimentos irradiados não prejudicarão a saúde em médio prazo.	LC	Q28. Işınlanmış gıdaların orta vadede sağlığa zarar vermediğini düşünüyorum. (Q28. I consider that irradiated foods are not harmful to health in the medium term.)
Q29. Eu considero que os alimentos irradiados não fazem mal à saúde a longo prazo. (Q29. I consider that irradiated foods are not harmful to health in the long term.)	Q29. Işınlanmış gıdaların uzun vadede sağlığa zarar vermediğini düşünüyorum.	Q29. Acredito que os alimentos irradiados não prejudicam a saúde em longo prazo.	UC	Q29. Uzun vadede ışınlanmış gıdaların sağlığa zarar vermeyeceğini düşünüyorum.	Q29. Acredito que os alimentos irradiados não prejudicarão a saúde em longo prazo.	LC	Q29. Işınlanmış gıdaların uzun vadede sağlığa zarar vermediğini düşünüyorum. (Q29. I consider that irradiated foods are not harmful to health in the long term.)
Q30. Eu considero que os alimentos irradiados não fazem mal à saúde das próximas gerações. (Q30. I consider that irradiated foods are not harmful to the health of future generations.)	Q30. Işınlanmış gıdaların gelecek nesillerin sağlığına zarar vermediğini düşünüyorum.	Q30. Acredito que os alimentos irradiados não prejudicam a saúde das gerações futuras.	UC	Q30. Işınlanmış gıdaların gelecek neslin sağlığına zarar vermeyeceğini düşünüyorum.	Q30. Acredito que os alimentos irradiados não prejudicarão a saúde da próxima geração.	LC	Q30. Işınlanmış gıdaların gelecek nesillerin sağlığına zarar vermediğini düşünüyorum. (Q30. I consider that irradiated foods are not harmful to the health of future generations.)
Q31. A Organização Mundial da Saúde (OMS) e a Organização das Nações Unidas (FAO/ONU) recomendam a irradiação de alimentos. (Q31. The World Health Organization (WHO) and the United Nations (FAO) recommend the irradiation of food.)	Q31. Dünya Sağlık Örgütü (WHO) ve Birleşmiş Milletler Gıda ve Tarım Örgütü (FAO/ONU), gıda ışınlamasını önermektedir.	Q31. A Organização Mundial da Saúde (OMS) e a Organização das Nações Unidas para Agricultura e Alimentação (FAO/ONU) recomendam a irradiação de alimentos.	UC	Q31. Dünya Sağlık Örgütü ve Birleşmiş Milletler ışınlanmış gıdaları tavsiye ediyorlar.	Q31. A Organização Mundial da Saúde e as Nações Unidas recomendam alimentos irradiados.	LC	Q31. Dünya Sağlık Örgütü ve Birleşmiş Milletler Gıda ve Tarım Örgütü, gıda ışınlamasını önermektedir.

* Version Assessment: UC (unchanged); LC (little changed); VC (very much changed); CC (completely changed).

**Table 3:** Confirmatory factor analysis (CFA) of the ASCIF-TR (*n* = 346).

Items	Mean (SE)	S ^1^	C ^2^	L ^3^	A ^4^	Cronbach’s α
**Factor S ^1^—Safety of Irradiated Foods (15 items)**						α = 0.955
Q29. Irradiated foods are not harmful to health in the long term.	3.052 (0.052)	0.748				
Q11. I would encourage consumption of irradiated foods.	2.908 (0.053)	0.690				
Q28. Irradiated foods are not harmful to health in the medium term.	3.231 (0.048)	0.815				
Q30. Irradiated foods are not harmful to the health of future generations.	3.075 (0.051)	0.777				
Q13. I would consume irradiated food because I know it is safe.	3.124 (0.051)	0.789				
Q12. I would consume irradiated foods because I know they cause no health damage.	3.139 (0.050)	0.777				
Q27. Irradiated foods are not harmful to health in the short term.	3.301 (0.050)	0.732				
Q15. I approve of consuming irradiated foods.	3.150 (0.050)	0.765				
Q26. I feel safe about consuming irradiated foods.	3.188 (0.051)	0.811				
Q10. I would be willing to pay more for irradiated foods.	2.801 (0.056)	0.638				
Q9. I would consume irradiated foods.	3.341 (0.045)	0.639				
Q21. I feel confident buying food labeled “processed by irradiation”.	3.260 (0.049)	0.737				
Q23. I would buy irradiated food knowing this process does not make it radioactive.	3.327 (0.051)	0.783				
Q25. Irradiated foods are safe from a nutritional standpoint.	3.257 (0.046)	0.797				
Q31. The WHO and FAO/UN recommend food irradiation.	3.263 (0.050)	0.735				
**Factor C ^2^—Concepts (8 items)**						α = 0.887
Q2. Food irradiation can be used to reduce microbial load.	3.618 (0.045)		0.728			
Q7. Food irradiation can extend the shelf life of foods.	3.642 (0.048)		0.671			
Q3. Food irradiation can inhibit sprouting of bulbs, roots, and tubers.	3.601 (0.045)		0.750			
Q5. The minimum absorbed dose must be sufficient to achieve the intended purpose.	3.587 (0.046)		0.732			
Q6. Türkiye authorizes the use of food irradiation.	3.439 (0.044)		0.613			
Q24. Irradiated foods are microbiologically safe.	3.361 (0.048)		0.532			
Q4. Food irradiation can delay the ripening of fruits.	3.538 (0.048)		0.641			
Q1. Irradiated food is different from radioactive food.	3.604 (0.047)		0.608			
**Factor L ^3^—Labeling (5 items)**						α = 0.912
Q18. Irradiated foods should clearly display this information on packaging.	3.766 (0.057)			0.836		
Q20. I consider the Radura symbol important on labels of irradiated foods.	3.697 (0.054)			0.785		
Q22. Food labels should prominently highlight irradiation information.	3.650 (0.052)			0.726		
Q19. Additional information on irradiated food labels is important.	3.763 (0.054)			0.843		
Q16. Educational campaigns are necessary to inform the public about food irradiation.	3.633 (0.052)			0.687		
**Factor A ^4^—Awareness (3 items)**						α = 0.778
Q14. I am aware of at least one irradiated food product.	3.107 (0.060)				0.692	
Q17. I know the Radura symbol used to represent irradiated food.	3.012 (0.068)				0.635	
Q8. I consciously consume irradiated foods.	3.095 (0.053)				0.538	

^1^ S = Safety of irradiated foods. ^2^ C = Concepts. ^3^ L = Labeling. ^4^ A = Awareness. SE = Standard Error. The values represent the item-rest Pearson correlation coefficients. The total scale Cronbach’s α was 0.963.

**Table 4:** Component correlation matrix of ASCIF factors (*n* = 346).

	S	C	L	A
S	1.000			
C	0.689	1.000		
L	0.552	0.650	1.000	
A	0.774	0.556	0.419	1.000

Sqrt(AVE): S = 0.751; C = 0.663; L = 0.778; A = 0.625. All inter-factor correlations were below the respective sqrt(AVE) values, supporting discriminant validity.

**Table 5:** Reliability and convergent validity summary of ASCIF subscales (*n* = 346).

Factor	Items	α	CR	AVE	Factor Loading Range
S ^1^—Safety	15	0.955	0.951	0.564	0.638–0.815
C ^2^—Concepts	8	0.887	0.861	0.440	0.532–0.750
L ^3^—Labeling	5	0.912	0.884	0.605	0.687–0.843
A ^4^—Awareness	3	0.778	0.655	0.391	0.538–0.692
Total Scale	31	0.963	–	–	0.532 – 0.843

^1^ S = Safety of irradiated foods. ^2^ C = Concepts. ^3^ L = Labeling. ^4^ A = Awareness. α = Cronbach’s alpha; CR = Composite Reliability (McDonald’s ω); AVE = Average Variance Extracted. Criteria: α ≥ 0.70, CR ≥ 0.70, AVE ≥ 0.50.

## Data Availability

The original contributions presented in this study are included in the article/Supplementary Material. Further inquiries can be directed to the corresponding author.

## Supplementary Materials

The following supporting information can be downloaded at: https://www.mdpi.com/article/10.3390/foods15132278/s1, File S1: ASCIF TR raw data.
